# Online information needs of cancer patients and their organizations

**DOI:** 10.3332/ecancer.2011.235

**Published:** 2011-11-09

**Authors:** C Maddock, I Lewis, K Ahmad, R Sullivan

**Affiliations:** 1 Tenovus, Gleider House, Ty Glas Road, Cardiff, CF14 5BD, UK; 2 Kings Health Partners Integrated Cancer Centre, IEO-ICC Centre for OncoPolicy, Section of Research Oncology, Bermondsey Wing, Guy’s Hospital, Great Maze Pond, London SE1 9RT, UK

## Abstract

Increasingly patients, relatives and carers are accessing health information via the internet. However, the health profession and people affected by cancer are becoming concerned with the quality of that information. A European survey was conducted under the auspices of the FP7 European Commission funded Eurocancercoms project^1^ during the period September 2010–March 2011. Its aim was to assess current online information needs of people with cancer particularly those who seek information using online social media technologies and the internet more broadly. A literature review was undertaken to gain a greater understanding of health seeking behaviour regarding cancer patients’ information needs and patient preferences for accessing different formats and media. This was used to inform the design and validation of online pan-European, multi-lingual questionnaires distributed via patient organizations and via specific Eurocancercoms partner organizations. This paper presents the results of this survey and suggests recommendations to be incorporated into the design of the online platform, ecancerHub, one of the intended outcomes of the Eurocancercoms project following this research. People want a wide variety of easy to find, easy to understand accurate information about cancer and how it is likely to impact on their everyday lives and on those close to them. They differ in the amount and detail of the information they would like and on their ability to identify quality information and understand it sufficiently to base their health-care decisions on. The majority of respondents raised the issue of quality of information and many requested recommendations of websites by the people who usually influence them most, the health professionals involved in their care.

## Background

The aim of this research was to assess current online information needs of people with cancer particularly those who seek information using new media technologies.

This paper reviews some of the current ways people affected by cancer are accessing the information they want online and some of the concerns raised regarding their ability to do this with confidence particularly regarding issues of quality and understandability. It goes on to make recommendations for the providers of online health information.

The online information needs survey took place from September 2010 to March 2011.

### Background literature

It is estimated that each year there are 10.9 million new cases of cancer worldwide and that over 24 million people worldwide are living with cancer [1,2]. Patients have better health-care outcomes when they are more informed about their disease, more involved with their treatment choices and more invested in their health care [3]. Information provided to cancer patients has been shown to relieve anxiety and help them make informed treatment decisions [4–6]. The amount and type of information individuals want varies according to their unique information needs which in turn can vary according to the disease, its stage, and the person’s age, culture and beliefs. Published data (in the western world at least) demonstrate unequivocally that patients with cancer want and need information about their condition [7] and that moreover information satisfaction is a significant predictor of quality of life [8]. As well as having differing information needs, individuals also differ in their preferences of how they want to receive that information [9].

Patients are becoming more active consumers of health-related information and are often encouraged and even expected to be well informed and take a more active role in obtaining medical information to support their medical decision-making [10]. Findings from the first Health Information National Trends Survey (HINTS-designed to provide data every 2 years on how US adults use the internet) indicated that patients are looking online as a first resort, prior to talking with their physicians and then turning to them for approval as to the quality of that information [11]. Respondents on the HINT Survey (between 2002 and 2008) reported that their trust in information from health-care professionals had increased while their trust in health information from the internet had decreased [12].

There are increasing concerns about (a) quality of information available [13], (b) ability of patients to identify sites providing quality information [14], (c) ability to interpret that information—health literacy [15] and (d) access to the often very specific nature of an individual’s information needs [16]. The difficulties for seekers of relevant health information are thus very apparent.

In 2001, Cline and Haynes estimated that there were more than 70,000 websites disseminating health information and in excess of 50 million people seeking health information [13].Various studies have shown that between 16% and 69% of patients with cancer use the internet for health information [17]. Cline and Haynes also highlighted increasing concerns about people’s ability to access good quality information online. This has been recognized in health systems across the United Kingdom for example and a statement from Anne-Toni Rodgers of the National Institute of Health and Clinical Excellence (NICE) in 2001 shows the extent to which they perceive their role ‘a vital part of our work…is to ensure that..(patients) have access to the same high quality information as their doctors’ [18].

A study by Nielsen-Bohlman *et al* in 2004 showed that nearly half of all American adults (90 million people) have difficulty in understanding and acting upon health information and this could result in patients taking medicines erratically and being unable to understand instructions like ‘take on an empty stomach’ [19]. Low health literacy can be a barrier to equitable access to health services and health outcomes. Providers of health information and resources online should therefore give greater consideration to ensuring accessibility to populations with low health literacy [20].

The opportunity for the internet to empower patients and consumers and support their ability to make informed health-care decisions is enormous, but due to the overall variable quality of information on the web, the potential to act upon erroneous information or cause greater confusion is an all too real possibility. This concern may be further compounded as for many health consumers the internet may be their sole source of health information [14]. In order to help consumers discriminate between websites it has been suggested that health professionals take a greater role in directing patients to sources of good quality health information including websites [21]. A range of tools for rating the quality of information on the net has also emerged and Wilson in 2002 developed a classification of these different approaches used in English language websites [22].

## Methods

An online questionnaire using ZAP survey (an online survey tool allowing the development of a survey via a web browser) was designed to elicit the views of those affected by cancer regarding their online information needs and information-seeking practice with a particular focus on:

- their health consumer status e.g. patient, carer/family, patient advocacy groups
- type of online information wanted
- how people check the reliability of online information
- what different sources (e.g. internet—including social media, printed material, health professionals) are used for getting and sharing information
- factors influencing web searching
- resources used and preferred

The questionnaire comprised initial demographic questions which were then followed by a series of questions, several using a 5-point Likert scale response format. Questions were phrased to include both positive and negative responses to lessen the risk of bias. It included an open ended question: ‘What other types of information would you like to access online?’ and allowed comments on other questions in the survey.

The questionnaire was approved by the independent Eurocancercoms ethics committee. It was reviewed and piloted by the Patient Advisory Committee (PAC) of the European Cancer Organization following translation into German, French, Spanish and Italian, and it was distributed on a European level. Links to the questionnaire were available from the Eurocancercoms website and from other project partner’s websites. The questionnaire was also sent out to patient and consumer organizations that had agreed via a PatientView survey to be contacted for Eurocancercoms activities. PatientView is an independent, global, research-and-publishing organization working with patients and health and social campaigning groups worldwide.

The online survey gave real-time summary graphs and reports plus the ability to drill down to individual responses. The questionnaire was also analysed using SPSS v 19.

This Eurocancercoms research was funded by an FP7 European Commission funded project, grant no. 230548.

## Results

A total of 476 people accessed the online survey, ∼70% of whom fully or partially completed it. No questions were compulsory. Representatives from over 20 countries completed the questionnaire. The majority of responses were from the United Kingdom (22.8%), Denmark (20.1%), Italy (18.4%), Germany (15.7%), Spain (8%) and France (5.5%).

There were 297 comments across all surveys from all three free text questions, with 62 specifically in response to ‘What type of information would you like to access online?’ Another key function of the survey was to gather a list of websites currently being used by the respondents and in particular their preferred resources. There were 869 responses to the two questions ‘What Websites do you use? and ‘What Websites would you recommend?’ A total of 129 unique websites were identified. This information was used to populate one of the resource areas of the pilot website under development by the Eurocancercoms partnership (ecancerHub–http://www.ecancerHub.eu).

The Spanish survey free text responses from the pilot survey were used in the qualitative analysis as the pilot survey was inadvertently distributed beyond the pilot testers and was completed by 36 respondents. The pilot survey versions were changed slightly to reflect comments made by the testers and so were not included in the main data analysis but we considered it valuable to include the additional free text responses.

### Summary profile of respondents

A summary profile of respondents is displayed in Table 1

Other background information

**Figure 1:**
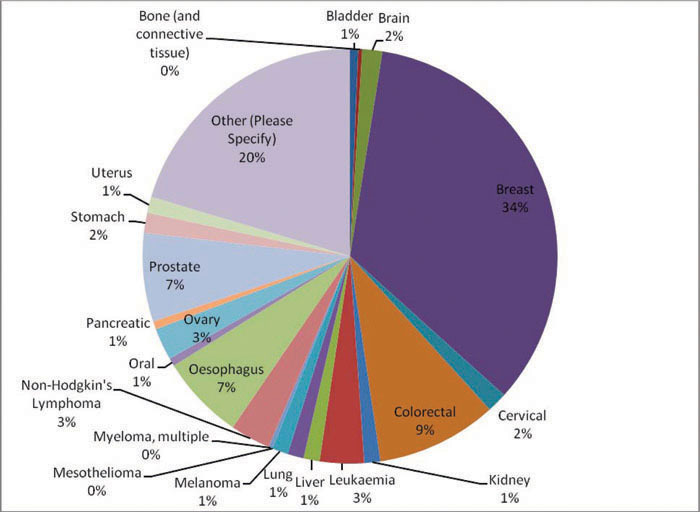
What type of cancer do you have?

- Only 11 respondents considered they belonged to an ethnic minority
- Respondents were from 24 countries in Europe. Main countries were United Kingdoms (22.8%), Denmark (20.1%), Italy (18.4%), Germany (15.7%), Spain (8%) and France (5.5%).
- The majority (82.9%) of respondents had a cancer diagnosis (over 20 types, Figure 1); 66.7% of whom were diagnosed within the last 5 years.
- Cancer types of those responding are displayed in Figure 1

### Main findings

**Figure 2:**
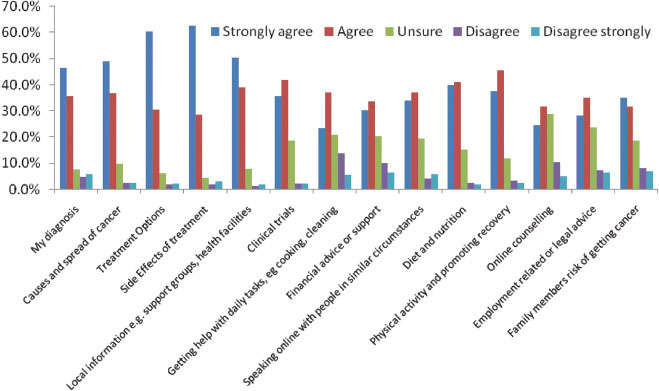
I want information on

Respondents wanted information on all aspects of cancer. Side effects and treatment options were the highest frequency responses for those who selected ‘strongly agree’ to the statement ‘I want information on…’. However, over 50% of respondents strongly agreed or agreed that they wanted information on all information suggested in the question (Figure 2). Other free text responses included: detailed, specific information on their own cancer type and an ability to ‘speak online’ and talk about experiences.

Findings regarding online information accuracy, searching behaviour and influence indicated that many respondents (55.7%) think that online health information is ‘mostly’ accurate although a not inconsiderable number (40.5%) thought it only occasionally or sometimes accurate (always accurate = 3.4%, never accurate = 0.4%). Most respondents (77.2%) strongly agreed or agreed that they would have more confidence in online health information if it was endorsed by a professional body. Most people (82.7%) strongly agreed or agreed that they search across several internet sites when looking for information. In a separate question, 62.4% strongly agreed or agreed that they focus on one trusted site. Having access to internet information does not appear to have considerably helped individuals to make treatment decisions, with a fairly even distribution across the responses. Having internet information has not, on the whole, added to the confusion about their condition and treatment options with 57.5% either strongly disagreeing or disagreeing that it had (Table 2).

**Figure 3:**
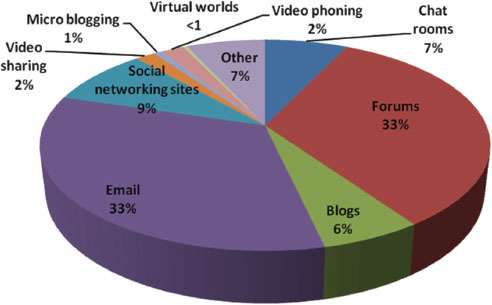
Have you ever used any of the following to talk about your cancer?

Participants use a variety of mechanisms to talk about their cancer online with the most popular mechanisms being: forums (33%) and emails (33%). Figure 3 shows the percentage of individuals that had used various online mechanisms for this purpose. Total numbers of respondents, *n* = 163. The most popular reasons given for accessing these mechanisms are ease of use (*n* = 112) and availability 24 hours a day (*n* = 96). More than one response could be selected.

**Figure 4:**
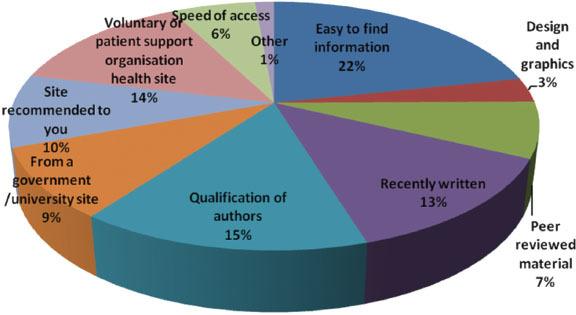
What factors influence your decision to look at or use a particular site?

The top five factors that influence respondents decisions to look at or use a particular website are easy to find information, qualification of authors, voluntary or patient support organization site, recently written and site recommended (Figure 4).

**Figure 5:**
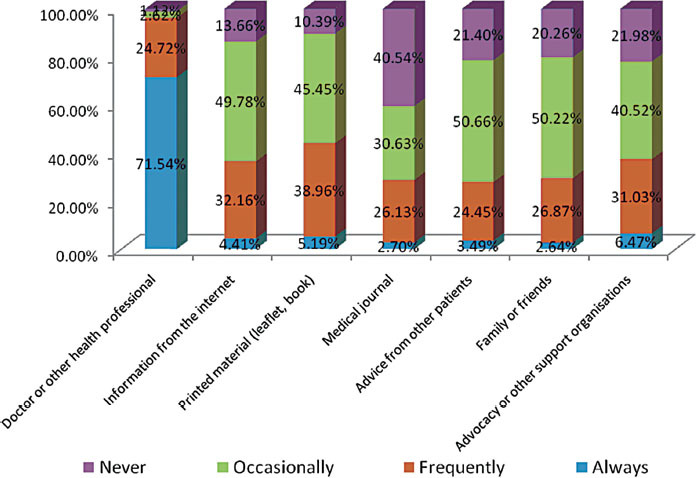
What or who influences your treatment decisions?

Unsurprizingly, doctors or other health professionals are most important in influencing patient’s treatment decisions, and 71.5% said they were always influenced by them and 24.7% were frequently influenced by them (Figure 5). Printed materials were slightly more influential (44.2% = always and frequently influenced) in respondents treatment decision-making than the internet (36.6%); advocacy or other support organizations had a similar influence (36.5% always or frequently influenced).

### Limitations

The survey was only translated into five of the main European languages: English, French, German, Italian and Spanish, and this is reflected in the number of responses from these countries. It is therefore impossible to advise that this research is representative of the views of those affected by cancer in the wider European community.

Although the survey was intended to be completed by individuals, one of the main mechanisms of sending out the survey was via patient organizations. In an accompanying email, we requested that this was either distributed or accessible to the organizations’ membership. Due to the nature of the survey and its planned anonymity, we are unsure as to whether this occurred or not.

Despite a particular push on the French survey by follow on emails to many French nonprofit cancer organizations, we still only had a small number of responses to the French survey.

Online surveys, particularly for researching online behaviours, may be advantageous in that they can be made available to large numbers of people in distant or isolated locations relatively simply. Lack of access to and/or familiarity of the internet may however limit the participation of already underrepresented groups in online survey research [23].

When attempting further offline analysis using the statistical package SPSS with the combined five language surveys, some anomalies with data categorization between text and numerical values were noticed. ZAP survey failed to respond to repeated requests for assistance or clarification. We were eventually satisfied with the data analysis but it was a more laborious and time-consuming process, which resulted in a more limited analysis than originally intended.

### Qualitative analysis of free text responses

A free response question ‘What other types of information would you like to access online?’ (Q 17) was included in the survey and 62 responses were received. Additional opportunities for comments in relation to the questions or more generic feedback elicited a total of 297 free text responses across all language surveys. As this data were reviewed it became apparent that it would be useful to analyse it further, as people had included comments that illustrated not only what other online information was wanted but why they wanted it (of the total responses, 137 were used in this analysis as other comments focussed on individual’s specific diagnoses or the questionnaire in general). A thematic qualitative analysis of the free text items moving beyond the listing of information types was undertaken manually. This consisted of an inductive and iterative process involving the identification of emerging themes and definition of a coding system. To increase the validity of the findings an independent researcher reviewed the categories to ensure agreement on the coding system.

The following categories of additional online information requested were identified from all five European language questionnaires completed (responses were translated where required). A selection of summarized comments and quotes are included below to highlight the main categories selected and to show that within these categories there exists a wide and sometimes opposing range of comments.

Main categories of information topics requested:

(a) General information
  1. Range of information (quantity/diversity)
  2. Format of information (simple/detailed /complex)
  3. Information to inform decisions
  4. Analytical skills needed to interpret information
  5. Amount or complexity of information causing anxiety
(b) Specific information
  1. Type of cancer: including treatment and side effects
  2. Services: including details of hospitals and clinicians and services such as information on local groups, social services etc.
(c) Support groups and psychological support from organizations and individuals in similar situations
(d) Rehabilitation and survivorship (maintenance of good health)
(e) Quality of information
  1. Credentials of site/author
  2. Recommendations from organizations or doctors
  3. Finding quality sites, up to date, reliable, accurate, evidence-based. Presence of poor quality sites and information
  4. Trust in Dr, trust in information on websites
(f) Sources of information
  1. Books/leaflets
  2. Face to face with Dr/other patients
  3. Internet

**Outliers**
(g) Not being informed by Drs/health professionals
(h) Different health-care systems

### General information

There were comments about the amount and detail of general information wanted (∼29% of all comments) with a further 48% on specific cancers, treatments or service-based information, generally with the proviso that the information is reliable and research based (23%). Just over a half of responders (56%) referred to the varying quantity and/or quality of information that can be found on the internet. There was recognition of the difficulties, particularly when having just been given a cancer diagnosis and possibly in an anxious or vulnerable state, of knowing where to look. It was important the way that the information is presented, for example ‘in a simple and non-threatening way.’ Respondents detailed differing information needs and how they may vary with time and circumstances.

It is clear from the responses to our survey that a large array of information was wanted and that the amount of detail requested varied equally. Approximately 29% of people wanted general information about cancer with about 9% of these specifying how they wanted the information to be presented for example an ‘easy to understand’ manner that would allow them to make decisions based on that information. There were 18 comments that specifically mentioned that the amount or complexity of information caused them anxiety or confusion, with a further seven comments highlighting the fact that analytical skills were required to interpret information.

There are many factors that can influence information need including patient’s age, sex, socioeconomic status and the period of time since diagnosis; many respondents recognized this when considering appropriate provision of information.

### General information


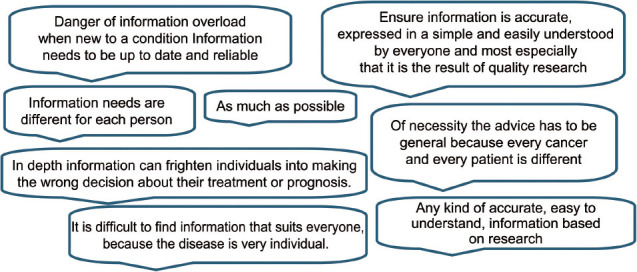
One comment expresses succinctly how information could be stepped from basic to detailed and includes a suggested range of topics.

‘Basic information that is clear, understandable, and that can lead onto, if required, more detailed and very specific information about all aspects of treatment, side effects, including long term, living with and beyond cancer including rehabilitation, diet, exercise etc’

### Specific information

As well as generic cancer information, about a third of respondents requested information on more specific cancer such as details of a particular cancer type (which could be subdivided further into diagnosis, treatment, side effects etc.). Practical signposting information such as where to find details about surgeons’ names, specialist treatment centres and their success rates, ranking or quality of services, and providers of services including information on voluntary organizations or support groups was requested by about 15% of responders.

### Specific cancer information


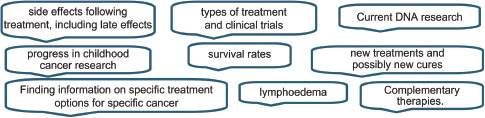


### Specific services


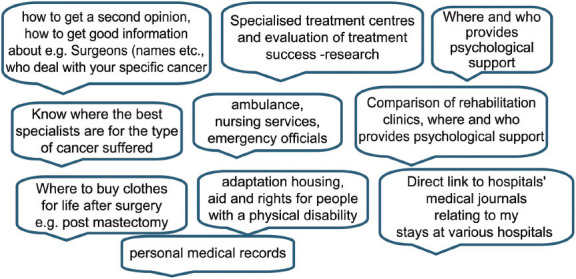


### Support groups (and psychological/emotional support)

Respondents expressed a need for detail on how to find and access support groups or voluntary organizations. The benefits of communicating with others in similar circumstances and/or the support given by voluntary organizations and support groups for all people affected by cancer were noted by over a quarter of responders. The comment below emphasizes the importance of speaking to others in similar circumstances while recognizing that others may prefer different mechanisms.

‘The discussion with fellow patients gives a more complete picture, it opens me up to the treatments, it makes life easier by using the experiences of others, but each patient is different.’

### Support groups (and psychological/emotionalsupport)


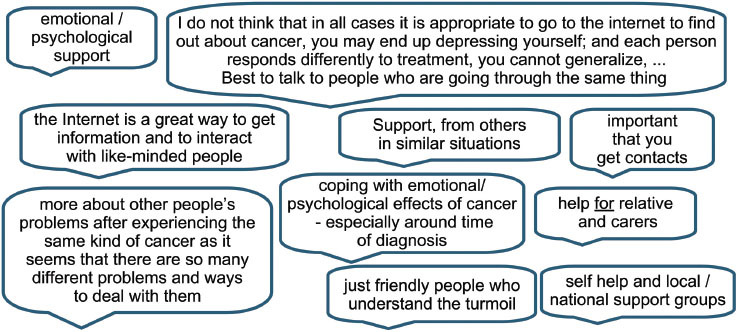


### Rehabilitation and survivorship

Rehabilitation and survivorship, including psychosocial issues were another key area where further access to information, advice and support was required (11%). 
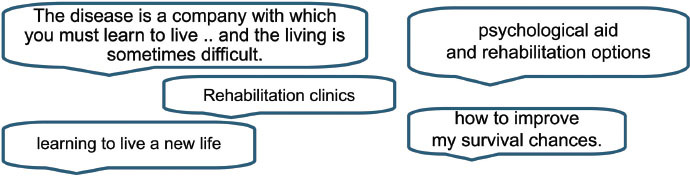


### Quality of information

Although having the right amount of information on a vast variety of topics, in an understandable format, was wanted, the quality of that information was essential. Of the 56% who mentioned the quality of information, 23% commented specifically on finding ‘reliable/quality accurate’ etc, sites and that indeed there were ‘good and bad’ sites out there. Several comments specified that the information allowed them to make informed decisions about their health-care choices and therefore, the validity of the information was of great consequence to them.

‘About my personal health, my diagnosis, my mode of treatment I can find no reliable information on the Internet. I can find additional and explanatory information on the net, I can influence my decisions about medicines but I have to constantly consult the doctor to advise me. You need reliable information re personal diagnosis to help influence decisions. The Internet is important for the first information and for explanations of technical terms, side effects, etc. forums are difficult to assess’

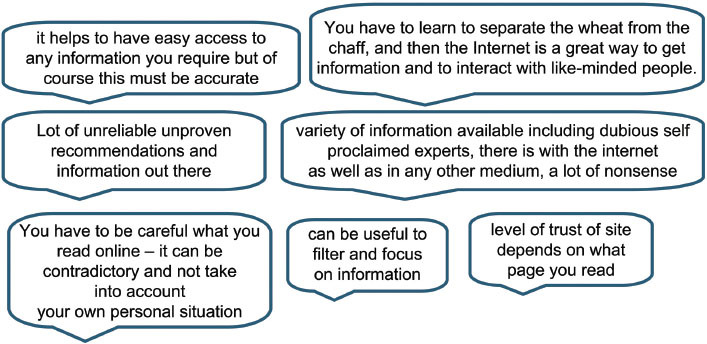


### Quality of information Finding ‘quality’/reliable sites

Nearly a quarter of responders considered that accessing quality sites could be more easily assured by either some form of accredited site, author credentials or by acquiring recommendations from doctors or reputable organizations. A few people commented on how they currently ensure they are accessing reliable information and others made suggestions who could take a role in raising awareness of respected and reliable sources. 
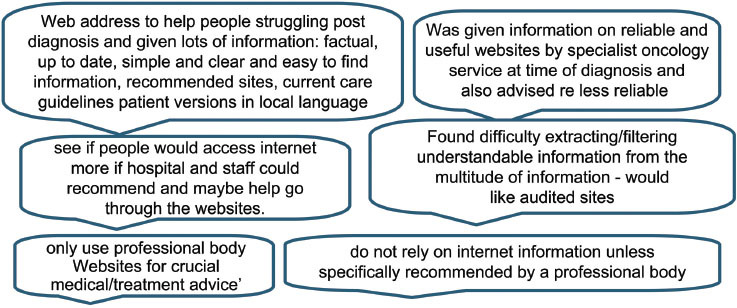


As stated earlier, a significant number of patients will at some point search online for information related to their cancer and the process could be less problematic if there was greater involvement of their health professionals in recommending reliable sites, or how to find them and secondly being prepared to discuss information sourced as described by this respondent below:

‘Some doctors encourage patients to use the internet and value the information which patients can use there. (There is a) Danger of people looking at unsubstantiated data from ‘rubbish’ sites. Some education of the patients might be helpful when they are first diagnosed with cancer; for example a booklet giving info on how to decide if a particular site was good and gave sound advice’

### Sources of information

It was found that respondents used different methods for finding the information they wanted. Some appeared to prefer one single route, such as speaking to their doctor only, while others used many different methods to achieve the level of information required for their needs. Information needs, it appeared, varied with time and circumstances and if the information was to aid a decision or fulfil an emotional need. Many people (34%) were happy to search for more general information on the internet but often wanted to speak face-to-face with health professionals or other patients (15%) when discussing their specific cancer information and treatment plans. A couple of people stated that they would like the ability to speak to a doctor or health professional online. The following comment describes one individual’s approach to information-seeking using a variety of sources to obtain sufficient information to ensure they were ‘happy’ with the outcome and able to use information to suit their purposes.

‘Having researched my condition I moved on to researching what options were open to me. When I had my list of available options I researched the advice of previous patients who had chosen the different options. I then weighed this information with that provided by my medical team. I was happy with this process as I felt involved with the decision making and was also able to ask sensible questions of my medics.’

### Sources of information


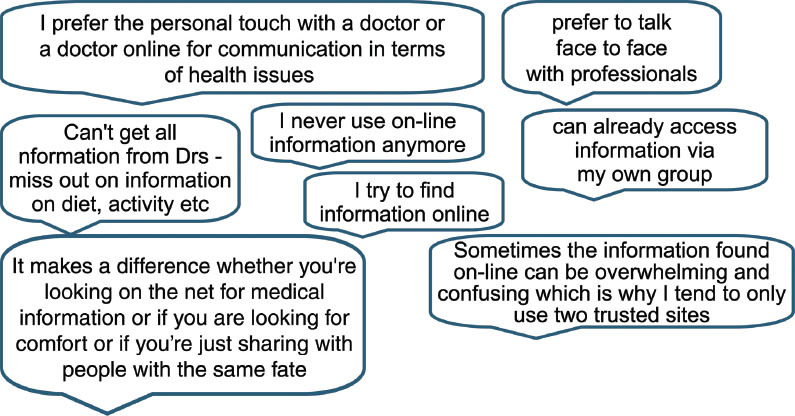


### Not being informed by Drs/health professionals

It was also apparent from some comments that health professionals do not always know about or do not recommend websites or organizations to their patients

‘It is appalling how many UK Oncologists have never heard of Dana Farber or MD Anderson! And as for Gustav Roussy or University of Jena - they haven’t a clue!’

‘I know there are many things to which I have drawn the attention of doctors, for example; I can apply for a disability permit; that I should have been granted at follow-up treatment…. the medical staff didn’t know about it.’

### Different systems in different countries

There was also acknowledgment from some [7] respondents that there were differences between countries health systems and from people who had experience of more than one country’s health-care provision and services and made comparisons between the two.

‘As a patient who has experienced British and European cancer treatment, I am sure our lower survival rate is because we are not helped with dealing with drug side effects.’

Also there could, correspondingly, be differing information requirements.

‘it is also relevant to consider country differences in medical protocol’

And a comment on behalf of Cancer Support France^2^, a French organization with a network of independent affiliated associations that have developed in recognition of the needs of English-speaking people affected by cancer.

‘Language difficulties pose huge problems, and much of our work consists of helping those who have very limited French skills understand what they are being told’

Conversely one Danish responder ‘did not want to exclude the possibility of receiving information from other sources than the strictly Danish sites’

A quote from one individual in particular succinctly sums up many of the requirements of the information wanted by patients:

‘When I was told I had cancer I was given a lot of information but it was a lot to take in at the time and when you are still struggling with the statement that you have cancer it makes the mind go a little blank, it would be a good idea to give the person a Web address where 1. they can go and have a look at. 2. This site must be simple and clear and be easy to find the different types of cancer. 3. It must then explain the different treatments (simple to read) 4. There should be provision to ask a question about your cancer. 5. MOST IMPORTANT It must be factual and updated often to show new drugs and treatments’.

## Discussion

The aim of this project was to review current online information needs of people affected by cancer and gain a greater understanding of health seeking behaviour and preferences for accessing different formats and media.

Analysis of the survey questions and free text responses reveals the variety of information wanted by people, from ‘basic information that is clear, understandable’ to ‘more detailed and very specific information about all aspects of treatment, side effects, including long term, living with and beyond cancer including rehabilitation, diet, after exercise etc.(quote from survey respondent).

Individuals may want to obtain this information from one or a variety of sources that can include their health professionals, speaking online or face to face with people with similar conditions and general searching online. It is also clear from our results that what people want is reliable information that can help them make decisions and choices about all aspects of their health care and daily living. Good quality information for patients about health problems can help in disease prevention, promotion of self-care, can inform treatment decisions and improve the effectiveness of clinical care [24].

The extent and variety of information types needed and delivery mechanisms suggested in our research can be broken down into the following broad areas:

- What information people want;
- Why they want the information; and
- Accessing information and understanding it

### What information people want

Results from the survey showed that people want a broad range of both clinical and nonclinical information. This resonates with a plethora of published research that demonstrate that most cancer patients want and need to be given all available information about their condition, good or bad [25] and the vast majority want a great deal of specific information about their illness and treatment [26]. A study by Davies *et al* in 2008 showed that 94% of patients wanted as much information as possible after their cancer diagnosis [27].

**Figure 6:**
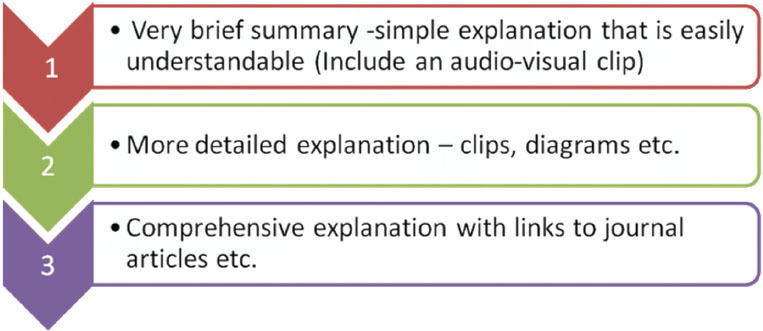
Stepped approach to information delivery

Some (13%) of our free text responses in particular however did suggest caution regarding the amount of potentially ‘overwhelming’ information available. A study exploring why some patients do not want additional information beyond a brief explanation by their physician found that all patients wanted basic information on diagnosis and treatment plans but then either (a) put their ‘faith’ in the doctors expertise, (b) wished to retain ‘hope’ and this was preserved by avoiding information or (c) fitted into a ‘charitable-charity’ attitude recognizing that scarce resources including information and time had to be shared out [28]. There will inevitably be some people who do not want information at all or a bare minimum and for those that live by the ‘ignorance is bliss’ approach, their wishes should be respected [29]. Information needs can also vary according to patients own attitudes and beliefs and may change during the course of their illness, so a stepped approach to information display should be considered (Figure 6)

In order to develop relevant, accurate, reliable and sufficiently detailed clinical information to help patients make treatment choices a more systematic approach to involving patients and other stakeholders in developing, evaluating and sharing patient information resources should be considered [24].

### Why people want online information

Patients and their families are often faced with complex information and treatment decisions. They may have to listen to and understand both their diagnosis and prognosis dependent on certain treatment options, evaluate additional information to aid their understanding (if they are fortunate, from a recommended source), compare the risks and benefits and articulate any concerns. They may need to ask pertinent questions, describe possible side effects and understand medical advice and treatment directions.

The central premise in the book ‘The Resourceful Patient’ is that future patients should accept responsibility for their own health care and try to become expert in managing their chronic illnesses by accessing online information, including many of the same resources that doctors do, and arranging their own care with the help of clinicians. Doctor’s expectation of their patients will be that they have responsibilities as well as rights and that they will make decisions with their doctors, be keepers of their own information and medical records on the web, will manage their condition and take care of their own simple illnesses [30].

Responses in our research revealed a requirement for information to empower and support people in discussions with their health-care professionals and also to identify tools to aid them in the decision-making process. Our research and earlier studies have shown that patients would like and can benefit from stories of other patients and that the internet is an important source of these stories [31]. Social media can allow people to benefit from the ‘collective wisdom’ of many other people [32,33]. Peoples shared stories and experiences can increase clinical knowledge and reveal how other people have managed their similar immediate health-care needs and long-term conditions as well as providing psychological support. ‘When patients managing the same chronic condition share observations with each other, their collective wisdom can yield clinical insights well beyond the understanding of any single patient or physician’ [32].

### Accessing information and understanding it

In general, people want to be able to search for information on the internet and to look for alternative sources of information and possibly clarification. Our results showed that most respondents search across several internet sites when looking for health information, though others rely mainly on one trusted source. Respondents agreed that a potential way of increasing their confidence in sites was their endorsement by professional bodies. However, few participants in research carried out by Eysenbach and Köhler in 2002 took notice of the website details or who was responsible behind the sites once they had found the information [34].

The majority of our survey respondents (55.7%) think that online health information is ‘mostly’ accurate but that still leaves 40.5% thinking it is only ‘occasionally’ or ‘sometimes’ accurate, and this may not give people the necessary confidence to base important decisions on. In a study by Marshall and Williams in 2006, patient support groups who were recruited to take part in an information review of a set of health information materials demonstrated lack of confidence in their ability to select quality health information and relied on preselection by authoritative sources such as libraries, support groups and health professionals and distrusted the internet [35]. Free text responses in our research included requests for health professionals to recommend websites. If health professionals were to offer guidance on suitable websites and more importantly why they make those recommendations then patients can begin to understand the rigorous standards applied to quality online sites and in medical and scientific journals and in contrast information sources with anecdotal or unsubstantiated postings. Health-care professionals may be best able to help patients by reviewing relevant websites in their areas of expertise and making specific recommendations to patients. At the University of Michigan Comprehensive Cancer Center, a mechanism for physician review of medical information on websites deemed appropriate to be recommended for patients has been designed. Health-care providers with expertise in specific areas review websites and sort them by specific diagnoses prior to listing them on the Cancer Center website as a source of additional information [3].

The sometimes transient nature of websites can lend support to the argument for empowering patients to adopt suitable strategies for evaluating health information rather than merely providing them with a list. Also some people may prefer to do their own research that extends beyond a list of recommended sites particularly where their views may diverge from those of their health-care providers. Comprehensive guidelines for evaluating web resources on health information are available, and these should be accessible and promoted to patients [22,36]. Alternatively, a simple checklist of advice could be displayed on websites and used to highlight the importance of using reliable sites.

Mechanisms to enable people to be more confident in accessing quality sites or that perform that function automatically were suggested by Wilson in 2002 [ref. 22], and include:

- Quality labels: A quality label (logo or symbol) represents a commitment by a provider to adhere to a code of conduct e.g. Health on the Net Foundation. (The Health On the Net Foundation (HON) promotes and guides the deployment of useful and reliable online health information. HON is a nonprofit, nongovernmental organization, accredited to the Economic and Social Council of the United Nations).
- User guidance: This enables users to check if a site complies with certain standards by answering a series of questions from a displayed logo e.g. DISCERN (DISCERN is a brief questionnaire which provides users with a valid and reliable way of assessing the quality of written information on treatment choices for a health problem.)
- Filtering tool: filters applied manually or automatically to accept or reject whole sites of information based on preset criteria
- Third party quality and accreditation labels: Third parties range from intraorganization bodies offering their services at low cost, similar to those responsible for the CE mark on electrical goods sold in the European Union, to high cost external independent assessors who perform audits and grant accreditation
- Codes of conduct for website developers: sets of quality criteria providing a list of recommendations for developing the content of a website e.g. eHealth Code of Ethics of the Internet Health

The value of developing and using rating systems and quality standards has been questioned by Delamothe in 2000 who argues that consumers will learn to cope with web content in spite of much of the information being incomplete or wrong [37]. This however can put what seems to be an unnecessary burden on people, particularly if seeking information at diagnosis when potentially in an anxious state and perhaps less inclined to compare sites, critically appraise, determine relevance and validity etc. Risk suggests that what could work is the automatic filtering out of websites that do not conform to ethical standards along with the ability to apply one’s own quality criteria. This would, he suggests, require advanced web browsers or a system of certification by a trusted and credible organization, with a well-known brand and that is recognizable worldwide [22]. Another way to address this issue is to provide consumers with tailored information that is contextualized and personalized e.g. directly relevant and easily comprehensible to the person’s own health situation. The approach proposed uses a theoretical framework of communication in order to support the consumer’s capacity to understand health-related web-based resources [15]. Search engines can provide consumers with a means for bypassing information that appears too technical or does not fit into their previous search behaviour or criteria, though this can result in omissions of information most relevant to their needs. Search technologies need to be more interactive to allow users to define their personal requirements, be context-driven, use the ‘collective wisdom’ of social media (e.g. a more refined version of people who searched for HER2-positive also looked for….). State-of-the-art search engine technologies are not currently widely available to those who can benefit most from them.

### Understanding health information

Ability to access quality health information, however, is ineffective if the information is not understandable to the consumer. There is a need to help bridge the gap between access to information and information understanding. A review of coverage of key information on English and Spanish language websites found that although the accuracy of the information provided was generally good, high reading levels were required to comprehend them [10]. Health literacy is defined broadly by the World Health Organization:

‘Health literacy represents the cognitive and social skills which determine the motivation and ability of individuals to gain access to, understand and use information in ways which promote and maintain good health. Health literacy means more than being able to read pamphlets and successfully make appointments. By improving people’s access to health information and their capacity to use it effectively, health literacy is critical to empowerment’ [38].

Examples of basic health information include the ability to understand instructions on prescription, drug bottles and consent forms, and the ability to negotiate complex health-care systems. According to the American Medical Association, poor health literacy is ‘a stronger predictor of a person’s health than age, income, employment status, education level and race’ [39,40].

## Conclusion and recommendations

People affected by cancer want a wide variety of accurate, easy to find and easy to understand information, and how it may impact on their lives and on those close to them. People differ in the amount, detail, how and when they would like to receive that information and also on their ability to identify quality information and understand it sufficiently to base treatment decisions on it.

The development of a website such as ecancerHub by experts across all oncological specialities as a dedicated and reliable site where people can be assured of its credentials (current, expert led, evidence based etc) seems a good place to start. This caters directly to those patients who would like to head straight to one reputable site, but also to those who strive to contribute to the collective wisdom and best practice of the wider cancer community worldwide by participating in online debate and sharing, for example, their own useful resources.

The improvement and relevance of such a site would be an ongoing venture by many key players. Knowledge advances, new evidence emerges, technologies and treatments are developed and correspondingly informational resources will need to change. Health-care professionals can additionally serve and support patients by playing an active and informed role in updating cancer information on the web.

## Figures and Tables

**Table 1:**
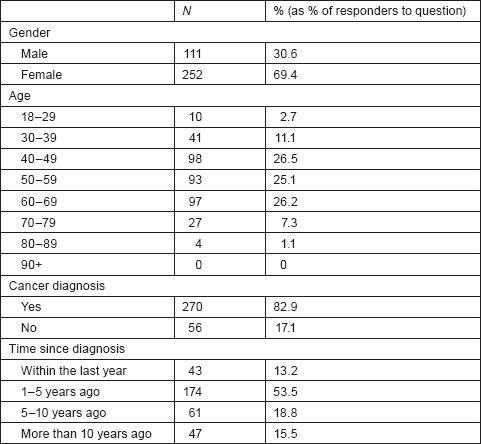
Respondents summary profile (gender, age, time since diagnosis).

**Table 2:**
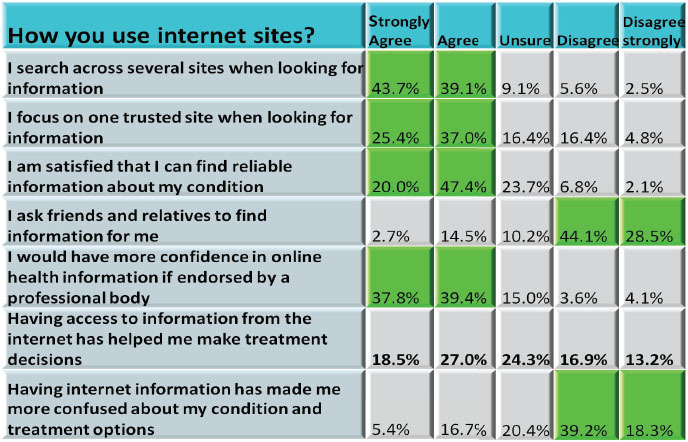
How you use internet sites

## Footnotes

2 Cancer Support France conducted a survey on ‘Language Support for Anglophone Cancer Patients Survey’, March 2010

## References

[1] Ferlay J, Shin HR, Bray F, Forman D, Mathers C, Parkin DM, GLOBOCAN 2008 (2010). Cancer Incidence and Mortality Worldwide: IARC CancerBase No 10 [Internet].

[2] CRUK. Cancer worldwide-the global picture (2008). http://info.cancerresearchuk.org/cancerstats/world/incidence/.

[3] Biermann JS (1999). Evaluation of cancer information on the Internet. Cancer.

[4] Ziegler L (2004). A literature review of head and neck cancer patients information needs, experiences and views regarding decision-making. European Journal of Cancer Care.

[5] Harris KA (1998). The informational needs of patients with cancer and their families. Cancer Practice.

[6] Rees CE, Sheard CE, Echlin K (2003). The relationship between the information-seeking behaviours and information needs of partners of men with prostate cancer: a pilot study. Patient education and counseling.

[7] Mossman J, Boudioni M, Slevin ML (1999). Cancer information: a cost-effective intervention. European Journal of Cancer.

[8] Davies N (2007). Empowering Cancer Patients to make Informed Decisions. Clinical Focus Cancer Medicine.

[9] Overberg R, Man Ade OW, Toussaint P, Westenbrink J, Zwetsloot-Schonk B (2010). How Breast Cancer Patients Want to Search for and Retrieve Information From Stories of Other Patients on the Internet: an Online Randomized Controlled Experiment. J Med Internet Res.

[10] Berland GK (2001). Health Information on the Internet. JAMA.

[11] Hesse BW (2005). Trust and sources of health information: the impact of the internet and its implications for health care providers: findings from the first health information national trends survey. Arch Intern Med.

[12] Hesse BW, Moser RP, Rutten LJ (2010). Surveys of physicians and electronic health information. New England Journal of Medicine.

[13] Cline RJW, Haynes KM (2001). Consumer health information seeking on the Internet: the state of the art. Health Education Research.

[14] Lorence DP, Greenberg L (2006). The Zeitgeist of Online Health Search. Journal of General Internal Medicine.

[15] Alpay L (2009). Current challenge in consumer health informatics: bridging the gap between access to information and information understanding. Biomedical Informatics Insights.

[16] Ankem K (2006). Factors influencing information needs among cancer patients: A meta-analysis. Library & Information Science Research.

[17] Huang GJ, Penson DF (2008). Internet health resources and the cancer patient. Cancer Investigation.

[18] Feinnman J (2001). Too much information?. The Observor Magazine.

[19] Nielsen-Bohlman L (2004). Health Literacy: A prescription to End Confusion.

[20] U.S. Department of Health and Human Services (2010). Health Literacy Online: A Guide to Writing and Designing Easy-to-Use Health Web Sites.

[21] Shepperd S, Charnock D, Gann B (1999). Helping patients access high quality health information. BMJ.

[22] Wilson P, Risk A (2002). How to find the good and avoid the bad or ugly: a short guide to tools for rating quality of health information on the internet / Commentary: On the way to quality. BMJ.

[23] Wright KB (2005). Researching Internet-based populations: Advantages and disadvantages of online survey research, online questionnaire authoring software packages, and web survey services. Journal of Computer-Mediated Communication.

[24] Coulter A, Ellins J, Swain D, Clarke A, Heron P, Rasul F, Magee H, Sheldon H (2006). Assessing the quality of information to support people in making decisions about their health and healthcare.

[25] Mesters I (2001). Measuring information needs among cancer patients. Patient Education and Counseling.

[26] Jenkins V, Fallowfield L, Saul J (2001). Information needs of patients with cancer: results from a large study in UK cancer centres. Br J Cancer.

[27] Davies NJ (2008). Information satisfaction in breast and prostate cancer patients: Implications for quality of life. Psycho-Oncology.

[28] Leydon GM (2000). Faith, hope, and charity: An in-depth interview study of cancer patients’ information needs and information-seeking behavior. West J Med.

[29] Thomas R, Thornton H, Mackay J (1999). Patient information materials in oncology: are they needed and do they work?. Clin Oncol (R Coll Radiol).

[30] Del Mar C, Muir Gray JA, Rutter H (2002). The resourceful patient.

[31] Deshpande A, Jadad AR (2006). Web 2.0: Could it help move the health system into the 21st century?. The Journal of Men’s Health & Gender.

[32] Sarasohn-Kahn J (2008). The wisdom of patients: health care meets online social media. iHealth Report.

[33] Suroweicki J (2005). The Wisdom of Crowds.

[34] Eysenbach G, Köhler C (2002). How do consumers search for and appraise health information on the world wide web? Qualitative study using focus groups, usability tests, and in-depth interviews. BMJ.

[35] Marshall LA, Williams D (2006). Health information: does quality count for the consumer?: How consumers evaluate the quality of health information materials across a variety of media. Journal of Librarianship and Information Science.

[36] Weber BA (2009). Educating patients to evaluate web-based health care information: the GATOR approach to healthy surfing. Journal of Clinical Nursing.

[37] Delamothe T (2000). Quality of websites: kitemarking the west wind. BMJ.

[38] Nutbeam D (1998). Health promotion glossary. Health Promotion International.

[39] Ad Hoc Committee on Health Literacy for the Council on Scientific Affairs, A.M.A. (1999). Health literacy. JAMA.

[40] AMA (1999). Health literacy. Report of the Council of Scientific Affairs. JAMA.

